# Supplementary material for: Visual perceptual learning of feature conjunctions leverages non-linear mixed selectivity
Source: NPJ Sci Learn. 2024 Mar 1;9:13. doi: 10.1038/s41539-024-00226-w (PMC10907723; doi:10.1038/s41539-024-00226-w)
Supplement: Supplementary file 1 — Supplementary material [file 41539_2024_226_MOESM1_ESM.pdf]

Supplementary information

Visual Perceptual Learning of Feature Conjunctions Leverages Non-linear Mixed Selectivity

Behnam Karami, Caspar M. Schwiedrzik

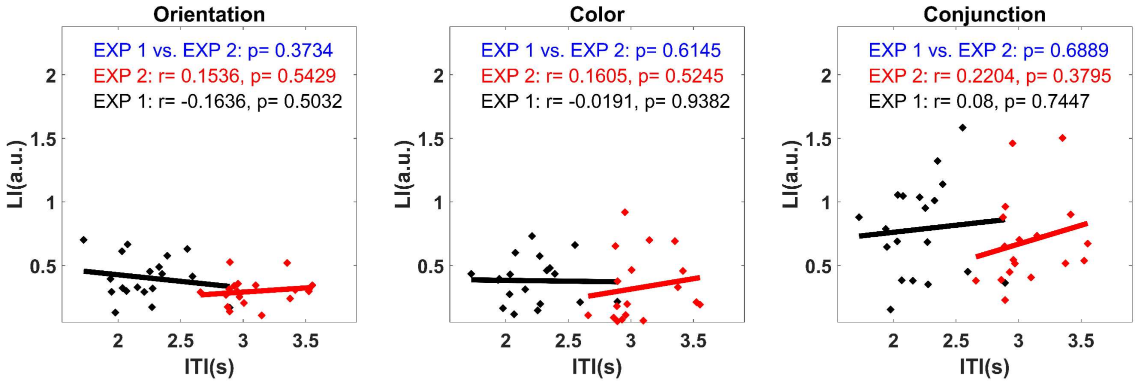

Supplementary Fig. 1 | Correlation between inter-trial-intervals (ITI) and Learning Indices (LI) for each feature and for the conjunction. Subjects self-initiated the trials, and there was between-subject variability in how much time they took to do so. **a,** Pearson's correlation between ITI and LI in the last training block (block 5) for each subject and each feature. There were no statistically significant correlations between ITI and LI for the features nor the conjunction, and no significant differences between Experiment 1 and Experiment 2. Statistics (*r* and *p*-values) are provided in the plot for each feature plus *p*-values for the comparison between Experiment 1 and 2 (in blue). Experiment 1 and 2 are color-coded black and red, consistent with the main text. The dots represent individual subjects and the lines represent a linear fit.

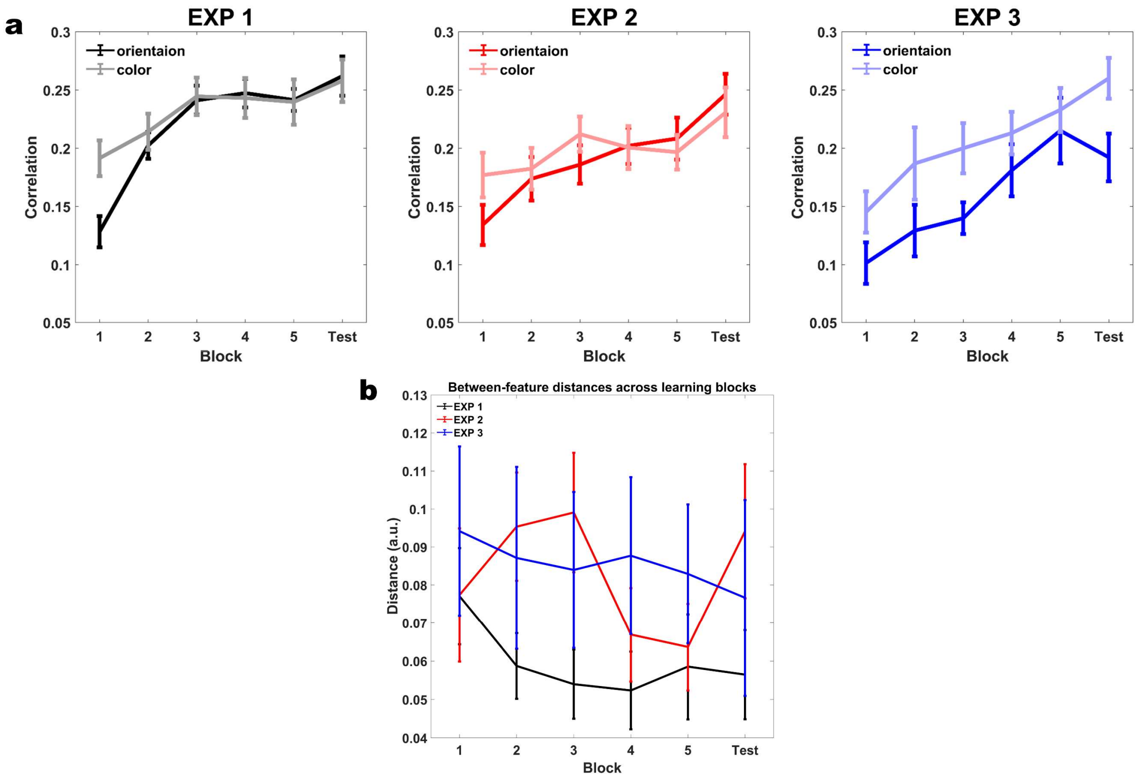

Supplementary Fig. 2 | Correlation between stimulus difficulty and error rate across training blocks. We hypothesized that an involvement of mixed selectivity representations in learning conjunctions should result in correlated errors between features, whereas independent purely selective representations should show no (or a lower) correlation between feature errors. To test this hypothesis, we measured Pearson's correlation coefficients between stimulus difficulty (inverse of stimulus strength, e.g., OD = 1,2,3 for OS = 3,2,1, respectively) and error rate for each feature and tracked it across training and the Test day. **a,** Pearson's correlation coefficient between stimulus difficulty and error rate per each feature across training and the Test day. Dark and pale lines represent orientation and color, respectively. As is evident in this plots, orientation and color correlation values converge and then highly overlap after block 2 most strikingly in Experiment 1. **b,** To further quantify this effect, we computed the distance between the two features' correlation values using inverse hyperbolic tangent ($tanh^{-1}$), as follows: $d = tanh^{-1}(ro) - tanh^{-1}(rc)$, where d is the distance between correlations, and ro and rc are the correlation values for orientation and color, respectively. Experiment 1 has significantly smaller distances across days than the other three Experiments ($F(2,45)=3.6934$, $p=0.0327$, $\eta^2=0.1410$). Experiment 1 was significantly different than Experiment 2 ($F(1,35)=5.9368$, $p=0.0201$, $\eta^2=0.1450$) and Experiment 3 ($F(1,33)=5.5295$, $p=0.0260$, $\eta^2=0.1649$). Experiments 2 and 3 did not differ significantly ($F(1,27)=0.0444$, $p=0.8348$, $\eta^2=0.0016$). These results suggest that information sources are getting unified as a result of learning under global feedback of Experiment 1. Black, red, and blue represent Experiments 1, 2, and 3 respectively. Error bars represent the standard error of the mean.
