# Supplementary material for: Visual perceptual learning of feature conjunctions leverages non-linear mixed selectivity
Source: NPJ Sci Learn. 2024 Mar 1;9:13. doi: 10.1038/s41539-024-00226-w (PMC10907723; doi:10.1038/s41539-024-00226-w)
Supplement: Supplementary file 2 — Reporting Summary [file 41539_2024_226_MOESM2_ESM.pdf]

nature portfolio

Corresponding author(s): Caspar M. Schwiedrzik

Last updated by author(s): Dec 14, 2023

Reporting Summary

Nature Portfolio wishes to improve the reproducibility of the work that we publish. This form provides structure for consistency and transparency in reporting. For further information on Nature Portfolio policies, see our Editorial Policies and the Editorial Policy Checklist.

Statistics

For all statistical analyses, confirm that the following items are present in the figure legend, table legend, main text, or Methods section.

n/a	Confirmed	
☐	☒	The exact sample size (*n*) for each experimental group/condition, given as a discrete number and unit of measurement
☐	☒	A statement on whether measurements were taken from distinct samples or whether the same sample was measured repeatedly
☐	☒	The statistical test(s) used AND whether they are one- or two-sided<br>*Only common tests should be described solely by name; describe more complex techniques in the Methods section.*
☐	☒	A description of all covariates tested
☒	☐	A description of any assumptions or corrections, such as tests of normality and adjustment for multiple comparisons
☐	☒	A full description of the statistical parameters including central tendency (e.g. means) or other basic estimates (e.g. regression coefficient) AND variation (e.g. standard deviation) or associated estimates of uncertainty (e.g. confidence intervals)
☐	☒	For null hypothesis testing, the test statistic (e.g. *F*, *t*, *r*) with confidence intervals, effect sizes, degrees of freedom and *P* value noted<br>*Give P values as exact values whenever suitable.*
☒	☐	For Bayesian analysis, information on the choice of priors and Markov chain Monte Carlo settings
☐	☒	For hierarchical and complex designs, identification of the appropriate level for tests and full reporting of outcomes
☐	☒	Estimates of effect sizes (e.g. Cohen's *d*, Pearson's *r*), indicating how they were calculated

Our web collection on statistics for biologists contains articles on many of the points above.

Software and code

Policy information about availability of computer code

Data collection	Psychtoolbox 3 of MATLAB R2019b was used to collect the data. Eye data was collected using SR Research Eyelink 1000+
Data analysis	The data was analyzed using custom codes written in MATLAB R2021b and R 4.2.1. The codes are available in figshare repository at .

For manuscripts utilizing custom algorithms or software that are central to the research but not yet described in published literature, software must be made available to editors and reviewers. We strongly encourage code deposition in a community repository (e.g. GitHub). See the Nature Portfolio guidelines for submitting code & software for further information.

Data

Policy information about availability of data

All manuscripts must include a data availability statement. This statement should provide the following information, where applicable:

- Accession codes, unique identifiers, or web links for publicly available datasets
- A description of any restrictions on data availability
- For clinical datasets or third party data, please ensure that the statement adheres to our policy

The data that support the findings of this study are openly available in figshare at .

Research involving human participants, their data, or biological material

Policy information about studies with human participants or human data. See also policy information about sex, gender (identity/presentation), and sexual orientation and race, ethnicity and racism.

Reporting on sex and gender	from 59 participants analyzed for this study, 39 were female, 20 were male.
Reporting on race, ethnicity, or other socially relevant groupings	Participants were not categorized in this study according to their race, ethnicity, etc. and such data was not collected from them since it was not in the main interest of the study.
Population characteristics	3 left-handed 56 right-handed, mean age 26 y/o, SD 4.16 y/o. Participants had no previous neurologic/psychiatric disorders
Recruitment	Participants aged 18 to 45 with no previous history of neurologic/psychiatric disorders with normal or corrected to normal vision were invited for the experiment. This invitation sent via an online recruitment system of the European Neuroscience Institute Göttingen (ORSEE) and hence, most of them were students or university employees. A brief D15 color vision test was performed to exclude subjects with impaired color vision.
Ethics oversight	All procedures were in accordance with the Declaration of Helsinki and approved by the Ethics Committee of the University Medical Center Göttingen (protocol number 29/8/17).

Note that full information on the approval of the study protocol must also be provided in the manuscript.

Field-specific reporting

Please select the one below that is the best fit for your research. If you are not sure, read the appropriate sections before making your selection.

☐ Life sciences ☒ Behavioural & social sciences ☐ Ecological, evolutionary & environmental sciences

For a reference copy of the document with all sections, see nature.com/documents/nr-reporting-summary-flat.pdf

Behavioural & social sciences study design

All studies must disclose on these points even when the disclosure is negative.

Study description	The data was behavioral responses of human participants in a visual task, hence, it was quantitative experimental.
Research sample	Participants aged 18 to 45 with no previous history of neurologic/psychiatric disorders with normal or corrected to normal vision were invited for the experiment. This invitation sent via an online recruitment system of the European Neuroscience Institute Göttingen (ORSEE) and hence, most of them were students or university employees (59 participants in total, 39 female, 3 left-handed, mean age 26 yrs, SD 4.16 yrs).
Sampling strategy	Participants were recruited randomly via an online application system. No sample size quantification was used. Sample sizes were determined according to previous studies of this type in the literature.
Data collection	Participant were trained on orientation and/or color discrimination tasks. The experiments took place over 3 to 5 consecutive days with one training session per day. Thus, training sessions were separated by one night of sleep. Stimuli were presented on an LCD monitor (ViewPixx EEG, refresh rate 120 Hz, resolution 1920 × 1080 pixel, viewing distance 65 cm) in a darkened, sound-attenuating booth (Desone Modular Acoustics). Stimulus delivery and response collection were controlled using Psychtoolbox (Brainard, 1997) running in Matlab (The Mathworks, Inc.) on Windows 10. During all experiments, we continuously acquired pupil and gaze measurements using a high-speed, video-based eye tracker (SR Research Eyelink 1000+). Participants had to report their choices with a saccade aimed at defined circular target positions on the screen.
Timing	The experiments took place over 3 to 5 consecutive days for each participant over a course of 10 months (Nov 2020 to Aug 2021).
Data exclusions	4 participants failed to improve throughout training and were thus excluded from the analysis.
Non-participation	No participant dropped out or declined participation.
Randomization	Participants were allocated randomly into four experimental groups.

Reporting for specific materials, systems and methods

We require information from authors about some types of materials, experimental systems and methods used in many studies. Here, indicate whether each material, system or method listed is relevant to your study. If you are not sure if a list item applies to your research, read the appropriate section before selecting a response.

Materials & experimental systems

n/a	Involved in the study
☒	☐ Antibodies
☒	☐ Eukaryotic cell lines
☒	☐ Palaeontology and archaeology
☒	☐ Animals and other organisms
☒	☐ Clinical data
☒	☐ Dual use research of concern
☒	☐ Plants

Methods

n/a	Involved in the study
☒	☐ ChIP-seq
☒	☐ Flow cytometry
☒	☐ MRI-based neuroimaging

Plants

Seed stocks

Report on the source of all seed stocks or other plant material used. If applicable, state the seed stock centre and catalogue number. If plant specimens were collected from the field, describe the collection location, date and sampling procedures.

Novel plant genotypes

Describe the methods by which all novel plant genotypes were produced. This includes those generated by transgenic approaches, gene editing, chemical/radiation-based mutagenesis and hybridization. For transgenic lines, describe the transformation method, the number of independent lines analyzed and the generation upon which experiments were performed. For gene-edited lines, describe the editor used, the endogenous sequence targeted for editing, the targeting guide RNA sequence (if applicable) and how the editor was applied.

Authentication

Describe any authentication procedures for each seed stock used or novel genotype generated. Describe any experiments used to assess the effect of a mutation and, where applicable, how potential secondary effects (e.g. second site T-DNA insertions, mosiacism, off-target gene editing) were examined.
